# PCBP1 is associated with rheumatoid arthritis by affecting RNA products of genes involved in immune response in Th1 cells

**DOI:** 10.1038/s41598-022-12594-7

**Published:** 2022-05-19

**Authors:** Xue Cao, Panlong Li, Xiaojuan Song, Lipu Shi, Lijie Qin, Dong Chen, Tianshu Chu, Yanwei Cheng

**Affiliations:** 1 Department of Rheumatology and Immunology, Henan Provincial People’s Hospital, People’s Hospital of Zhengzhou University, People’s Hospital of Henan University, Zhengzhou, China; 2 Department of Emergency, Henan Provincial People’s Hospital, People’s Hospital of Zhengzhou University, People’s Hospital of Henan University, Zhengzhou, China; 3 Wuhan Ruixing Biotechnology Co., Ltd, Wuhan, China

**Keywords:** RNA splicing, Immunological disorders

## Abstract

Rheumatoid arthritis (RA) is an autoimmune disease characterized by persistent synovitis, in which T helper 1 (Th1) can promote the development of a pro-inflammatory microenvironment. Poly(rC)-binding protein 1 (PCBP1) has been identified as a promising biomarker of RA, while its molecular mechanisms in RA development are unknown. As a canonical RNA binding protein, we propose that PCBP1 could play roles in RA by affecting both expression and alternative splicing levels in Th1 cells. Here, microarray datasets (GSE15573 and GSE23561), including 102 peripheral blood mononuclear cell samples from 39 RA patients and 63 controls, were used to evaluate the *PCBP1* expression changes in RA patients. High throughput sequencing data (GSE84702) of iron driven pathogenesis in Th1 cells were downloaded and reanalyzed, including two *Pcbp1* deficiency samples and two control samples in Th1 cells. In addition, CLIP-seq data of PCBP1 in Jurkat T cells was also analyzed to investigate the regulatory mechanisms of PCBP1. We found *PCBP1* were down-regulated in RA specimens compared with control. The result of differentially expressed genes (DEGs) showed that *Pcbp1* silencing in Th1 cells affected the expression of genes involved in immune response pathway. Alternative splicing analysis also revealed that PCBP1-regulated alternative splicing genes (RASGs) were enriched in TNF-a/NF-κB signaling pathway, T cell activation, T cell differentiation and T cell differentiation associated immune response pathways, which were highly associated with RA. DEGs and RASGs by *Pcbp1* deficiency in mice were validated in PBMCs specimens of RA patients by RT-qPCR. Investigation of the CLIP-seq data revealed PCBP1 preferred to bind to 3′UTR and intron regions. PCBP1-bound genes were also significantly associated with RASGs, identifying 102 overlapped genes of these two gene sets. These genes were significantly enriched in several immune response related pathways, including myeloid cell differentiation and positive regulation of NF-κB transcription factor activity. Two RA-related genes, *PML* and *IRAK1*, were screened from the above immune related pathways. These results together support our hypothesis that PCBP1 can regulate the expression of genes involved in immune response pathway, and can bind to and regulate the alternative splicing of immune response related genes in immune T cells, and ultimately participate in the molecular mechanism of RA, providing new research ideas and directions for clinical diagnosis and treatment.

## Introduction

Rheumatoid arthritis (RA) is a chronic immune-mediated disease characterized by persistent synovial inflammation that leads to organ damage predominantly in the joints^1–3^. Despite continuous progress in treatment over the past two decades, unfortunately RA remains an almost lifelong process that causes joint damage, disability, decreased quality of life, and cardiovascular and other comorbidities^3^. Annually, 5–50 per 100,000 individuals are affected by RA in industrialised countries^4, 5^. The immunopathogenesis of RA is a very complex process that involves genetic, epigenetic and environmental factors, but remains to be fully elucidated^4^. In fact, it is at least partially caused by the disequilibrium of iron homeostasis, which is considered to play a vital role in remodeling immune function and inflammatory response^6^. It is now established that iron deposition is observed in the synovial fluid from patients with RA^7–9^. Several studies involving mice models and human samples have also demonstrated some improvement in RA activity with treatment with iron-chelating agents^10–12^.

As an iron chaperone, poly(rC)-binding protein 1 (PCBP1) is a critical mediator for maintaining iron homeostasis by binding to iron and delivering it to ferritin and other iron-dependent proteins in mammalian cells^13, 14^. It is evident from the studies on autoinflammatory diseases and cancers that PCBP1 participates in the regulation of immune response. Ansa-Addo et al.^15^ found that PCBP1 functioned as a global regulatory node that subverts immunosuppression in cancer by modulating the balance between regulatory T cells (Tregs) and effector T cells, which are well-known regulators involved in the pathogenesis and development of RA^16^. In addition, Liao et al.^17^ provided experimental evidence that PCBP1 could modulate the innate immune response by facilitating the binding of Cyclic GMP-AMP synthase to DNA, which plays an important role in the innate antiviral response. In a recently published study^18^, PCBP1 was proven to promote granulocyte–macrophage colony-stimulating factor (GM-CSF) production produced by T helper 1 (Th1) cells, which is indispensable for the pathogenesis of many autoimmune diseases, including RA^19^. Of note, Xia and his colleagues^20^ conducted a multi-dataset analyses, in which seven microarray gene expression datasets representing various RA-related tissues/cells were analysed, and found that PCBP1 were connected with previously known RA genes. More importantly, the protein levels of PCBP1 showed significantly differentially expressed levels in RA patients compared with healthy controls. With these data, it is believed that PCBP1 may play pivotal roles in the pathogenesis of RA and could be a promising biomarker for RA. However current understanding on the roles of PCBP1 in RA remains unknown.

As is well known, PCBP1 is initially identified as an RNA binding protein (RBP) that participates in multiple gene regulatory levels, including gene transcription, post-transcription and translation. Recently, PCBP1 have been implicated in alternative splicing^21, 22^. Alternative splicing is a ubiquitous regulatory mechanism of gene expression that allows generation of more than one unique mRNA species from a single gene^23^, and has been shown to contribute to the development and progression of RA^24, 25^. However, there have been no studies to investigate the molecular mechanism on how PCBP1 participates in RA, and whether PCBP1 can affect RA via regulating expression and alternative splicing levels of genes, which may impede further study on complex molecular mechanism of this lifelong disease. RA is known to be a Th1-mediated autoimmune disease^26^. Herein, we aim to (1) evaluate the expression changes of *Pcbp1* in RA patients based on two public microarray datasets; (2) profile the differentially expressed genes (DEGs) in *Pcbp1*-knockdown Th1 cells based on a public RNA-seq data; (3) analyze the PCBP1-regulated alternative splicing events in Th1 cells; (4) validate DEGs and PCBP1-regulated alternative splicing events in PBMCs specimens from RA patients; (5) identify PCBP1 binding targets in Th1 cells based on a public CLIP-seq data; (6) reveal how PCBP1 modulates alternative splicing in Th1 cells via integrating *Pcbp1*-knockdown RNA-seq and PCBP1 CLIP-seq datasets. These findings will provid a comprehensive understanding on the involvement of PCBP1 in RA and a new research ideas and directions for clinical diagnosis and treatment of RA.

## Results

### Down-regulated Pcbp1 levels in RA patients

**Figure 1:**
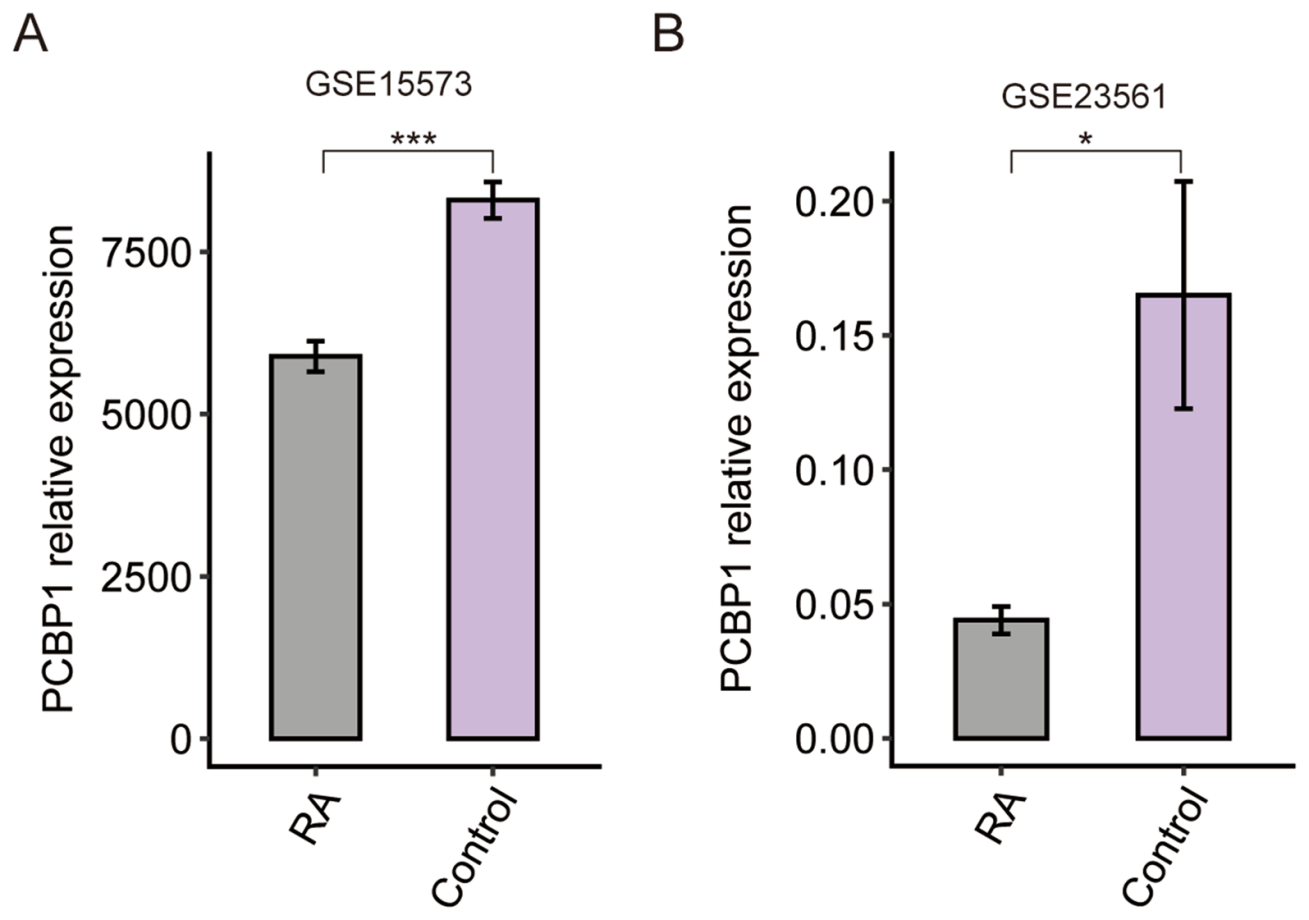
Relative expression levels of *Pcbp1* in RA patients and controls. Box plot showed the relative expression levels of *Pcbp1* in the microarray datasets of GSE15573 (**A**) and GSE23561 (**B**) used in this study.

Previous study reported that PCBP1 is a promising biomarker for RA^20^. To investigate the expression changes of *PCBP1* in RA patients, we downloaded the two published microarray datasets that GSE15573 and GSE23561, which contain 63 control-PBMCs samples and 39 RA-PBMCs samples. After reannotating probe ID into human gene symbols, the relative expression levels of *Pcbp1* were obtained. We found that *Pcbp1* were significantly down-regulated in RA specimens compared with control (Fig. 1A,B), indicating that PCBP1 was associated with RA and might play crucial roles in the development and progression of RA.

### Transcriptome profiles analysis between PCBP1-knockdown and control Th1 cells.

**Figure 2:**
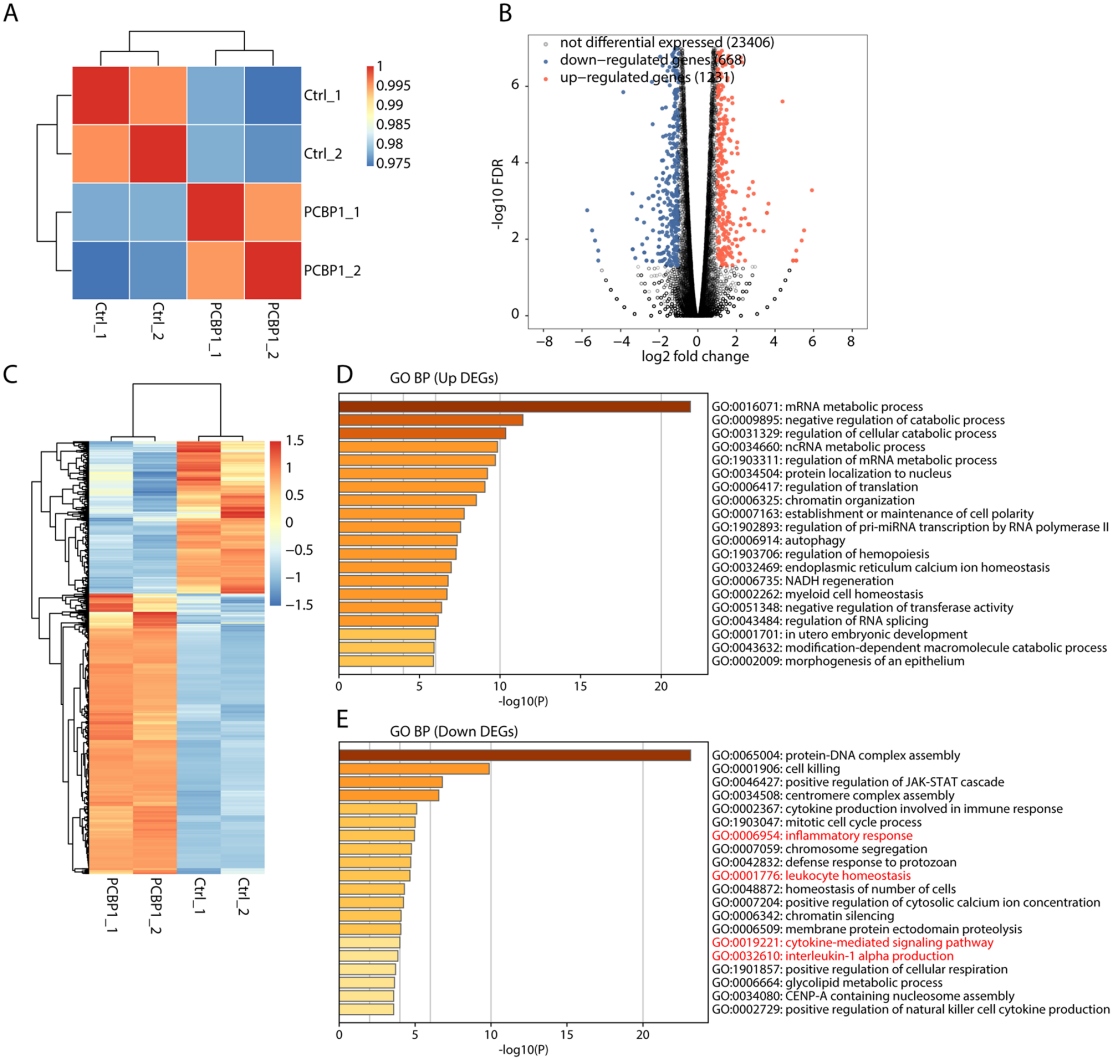
Transcriptome analysis of differentially expressed genes (DEGs) in *Pcbp1*-knockdown Th1 cells and control Th1 cells. (**A**) Hierarchical clustering heat map showed correlation between *Pcbp1*-knockdown and control samples based on FPKM value of all expression genes. (**B**) Volcano plot showed all DEGs between *Pcbp1*-knockdown and control samples with edgeR. FDR < 0.05 and FC (fold change) ≥ 2 or ≤ 0.5. (**C**) Hierarchical clustering heat map showed expression levels of all DEGs. (**D**) Bar plot exhibited the most enriched GO biological process results of the up-regulated DEGs, that making use of analyses by Metascape. (**E**) Bar plot exhibited the most enriched GO biological process results of the down-regulated DEGs, that making use of analyses by Metascape.

To decipher the regulatory functions of PCBP1 in RA, we also downloaded and re-analyzed published dataset (GSE84702), including two *Pcbp1*-knockdown (KD) and two control samples in mouse Th1 cells. After aligning quality filtered reads to the mouse genome (GRCm38), we obtained the expression level of all expressed genes and calculated the normalized FPKM (fragments per kilobase per million) values for them. Sample correlation analysis of the four RNA-seq samples revealed that *Pcbp1*-KD group were clearly separated from control group (Fig. 2A), indicating the global alteration of transcriptome by *Pcbp1*-KD. We then obtained the differentially expressed genes (DEGs) using edgeR package. Setting FDR < 0.05 and fold change > 2 as the criteria, 1231 up-regulated DEGs and 668 down-regulated DEGs were obtained between *Pcbp1*-KD *vs.* control (Fig. 2B). Hierarchical clustering heat map of the total DEGs showed a high consistent expression pattern in the two replicates, suggesting the high quality of RNA-seq data and DEG results (Fig. 2C). We then dedicated to explore the functions of these DEGs. By performing GO analysis, we found the up-regulated DEGs were enriched in metabolic or catabolic related terms, including mRNA metabolic process, negative regulation of catabolic process, and regulation of cellular catabolic process (Fig. 2D). For down-regulated DEGs, we found they were enriched in inflammatory response related terms, including cytokine production involved in immune response, inflammatory response, cytokine-mediated signaling pathway, and interleukin-1 alpha (IL-1α) production (Fig. 2E), suggesting that PCBP1 was highly associated with immune and inflammatory response in Th1 cells. We also analyzed the enriched Kyoto Encyclopedia of Genes and Genomes (KEGG) and Reactome pathways for DEGs. Up-regulated DEGs were highly enriched in spliceosome and mRNA splicing pathways (Fig. S1A,C). Down-regulated DEGs were enriched in immune and inflammatory response related pathways (Fig. S1B,D).

### PCBP1 globally modulates alternative splicing of genes in Th1 cells

**Figure 3:**
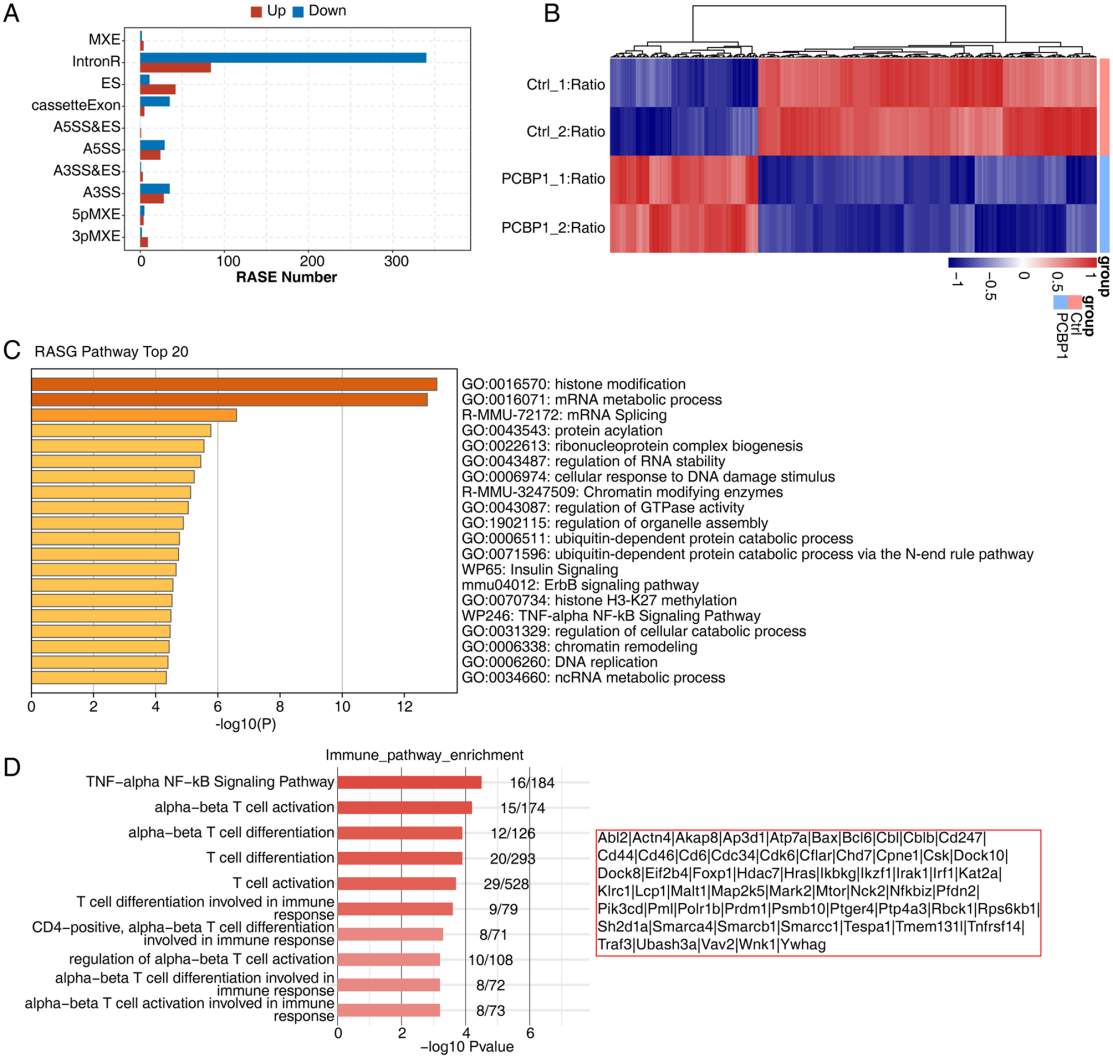
Transcriptome analysis of alternative splicing regulation in *Pcbp1*-knockdown and control samples. (**A**) The bar plot showed the number of all significant regulated alternative splicing events (RASEs). X-axis: RASE number. Y-axis: the different types of AS events. (**B**) Hierarchical clustering heat map of all significant RASEs based on splicing ratio. AS filtered should have detectable splice junctions in all samples and at least 80% samples should have >  = 10 splice junction supporting reads. (**C**) Bar plot exhibited the most enriched pathways results of the regulated alternative splicing genes (RASGs), that making use of analyses by Metascape. (**D**) Bar plot exhibited the most enriched immune pathways results of the regulated alternative splicing genes (RASGs),and the list of genes was on the right.

We then analyzed the regulated alternative splicing events (ASEs) by *Pcbp1*-KD in Th1 cells. We used ABLas pipeline to screen out ASEs and then identified the significantly PCBP1-regulated ASEs (RASEs) by Student’s *t*-test method. For all the detected ASEs, IntronR was the most detected type (Fig. S2A). We then calculated PCBP1-regulated ASEs (PCBP1-RASEs) and detected 2128 RASEs with *P*-value < 0.05. By classifying these RASEs into ten AS types, we found PCBP1-RASEs showed an inclination to several types. IntronR, exon skipping (ES), cassette exon, A3SS, and A5SS were the top five types (Fig. 3A). Meanwhile, much more ES events were promoted in *Pcbp1*-KD samples (up-regulated), and cassette exon events (opposite to ES) showed contrary pattern (down-regulated), suggesting that PCBP1 has the function to keep exons with transcripts during alternative splicing progression (Fig. 3A). If we regard IntronR as a specific cassette exon event, the much higher downregulation of IntronR also support the hypothesis that PCBP1 could retain sequences in transcripts during alternative splicing progression (Fig. 3A). The alternative splicing ratio heat map revealed that the detected RASEs showed a high consistency of the two replicates of control and *Pcbp1*-KD samples (Fig. 3B). We then extracted PCBP1-RASGs and performed functional enrichment analysis using Metascape platform. Top 20 enriched terms/pathways were shown in Fig. 3C. Histone modification, mRNA metabolic process, mRNA splicing, protein acylation, and ribonucleoprotein complex biogenesis were the top five enriched terms/pathways (Fig. 3C). Previous study^18^ demonstrated *Pcbp1* deficiency could inhibit pro-inflammatory cytokine expression in Th1 cells by inhibiting GM-CSF production and affecting *Csf2* RNA stability, we want to explore whether PCBP1 regulates inflammatory through alternative splicing regulation. We then extracted immune related pathways from RASG enriched pathways and found T cell activation and differentiation pathways were the most abundant (Fig. 3D). Detail gene symbols of these pathways were shown in the right panel of Fig. 3D, including *Irf1*, *Hdac7*, *Ptger4*, *Tespa1*, *Cbl*, and *Pml*. Interaction analysis between DEGs and RASGs showed that 146 genes were regulated at both expression and alternative splicing by *Pcbp1* deficiency (Fig. S2B). These results together demonstrated PCBP1 had the function to regulate alternative splicing of genes involved in immune response in Th1 cells.

### Identification of PCBP1 binding targets in Jurkat T cells

**Figure 4:**
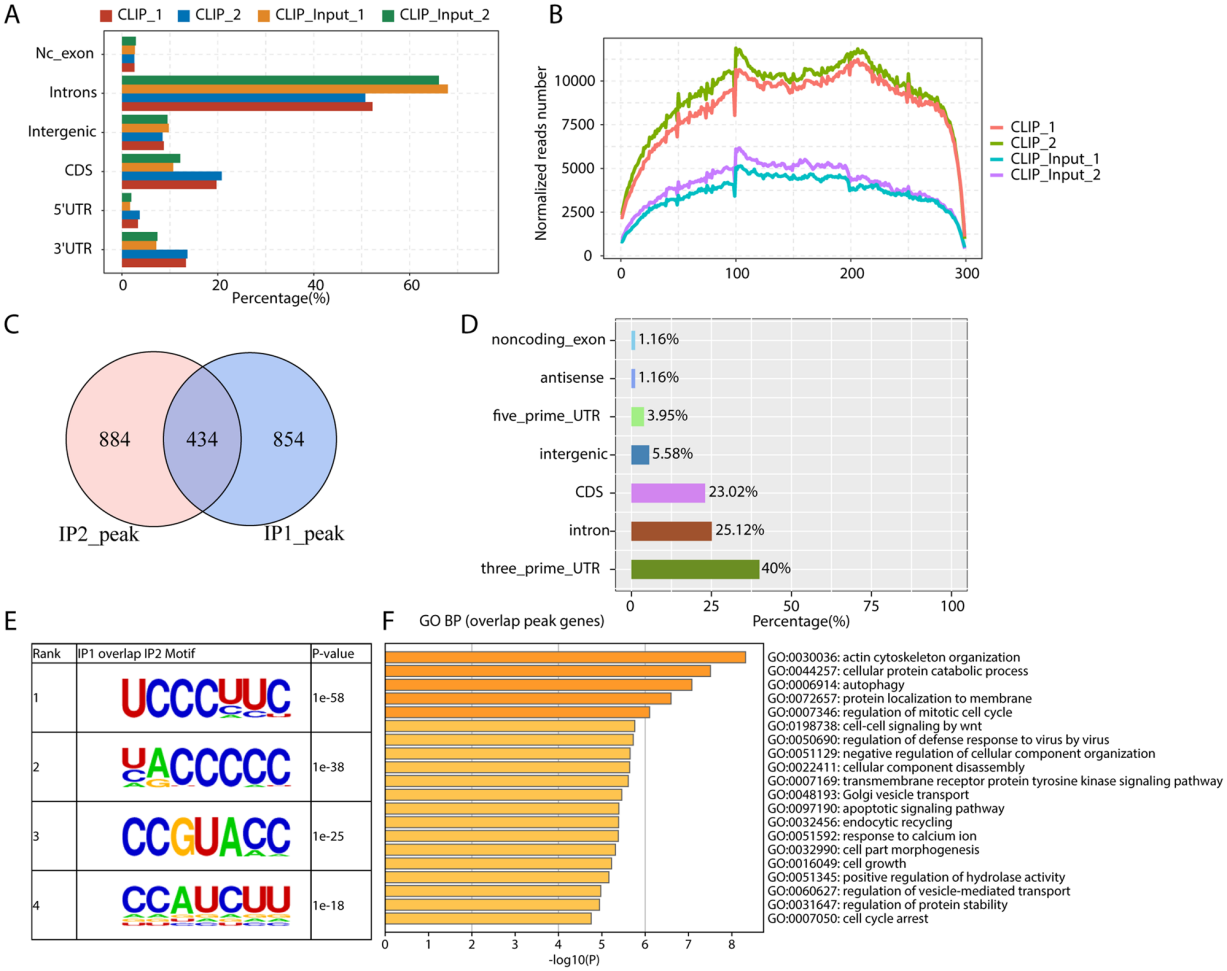
CLIP-seq analysis revealed the RNA binding features of PCBP1 in T lymphoma cell line. (**A**) Bar plot showed the reads distribution across reference genome. (**B)** Peak reads density in 5′UTR, CDS and 3′UTR. These three regions of each gene were separated into 100 bins, the UPF1 peak reads in each bin was calculated. The reads density of UPF1 peaks in all genes were plotted. (**C**) Venn diagram showed the overlap peaks in two IP samples. (**D**) Bar plot showed the overlap peaks reads distribution across reference genome. (**E**) Motif analysis showed the top 5 overlapped peaks preferred bound motifs of PCBP1 by HOMER software. (**F)** Bar plot exhibited the most enriched GO biological process results of the overlap PCBP1-bound genes, that making use of analyses by Metascape.

To further explore how PCBP1 regulates alternative splicing in Jurkat T cells, we downloaded and analyzed the sequencing data of CLIP-seq from the same study of *Pcbp1* deficiency RNA-seq data^18^ to investigate the RNA binding profile of PCBP1. After aligning quality filtered reads of the two replicates (CLIP_1 and CLIP_2) to the human genome (GRCh38), we analyzed the genomic distribution of aligned reads. Intronic region was the largest part of aligned reads for both PCBP1 and input samples, suggesting PCBP1 binds to pre-RNAs that are not fully spliced (Fig. 4A). PCBP1-bound reads were enriched in 5′UTR, CDS (coding sequence) and 3′UTR regions compared with input samples, while input samples were enriched in intronic region (Fig. 4A). The higher distribution of PCBP1-bound reads in exon regions could also be observed by accumulating aligned reads to the 5′UTR, CDS, and 3′UTR regions of all genes (Fig. 4B). We then performed peak calling analysis and obtained 1294 and 1325 PCBP1 binding sites from CLIP_1 and CLIP_2 samples, respectively. After merging overlapped genes from CLIP_1 and CLIP_2 samples, we found 434 merged peaks were both detected from the two replicates (Fig. 4C). Genomic distribution analysis of the overlapped peaks revealed they were mainly in 3′UTR, intron, and CDS regions (Fig. 4D). Motif analysis of the overlapped peaks revealed they were enriched in CU-rich elements (Fig. 4E). We also performed motif analysis for separate PCBP1-bound peaks of the two replicates, and found the CU-rich motifs were among the top five motifs for both replicates (Fig. S3A,B). Functional enrichment analysis of the overlapped peak genes showed the top 20 terms/pathways in Fig. 4F, including actin cytoskeleton organization, cellular protein catabolic process, autophagy, protein localization to membrane, and regulation of mitotic cell cycle as the top five terms (Fig. 4F). Other pathways, including protein metabolism, mitotic regulation, apoptosis and virus defense, were also enriched (Fig. 4F).

### PCBP1 modulates splicing of genes involved in immune response through binding to their pre-mRNAs

**Figure 5:**
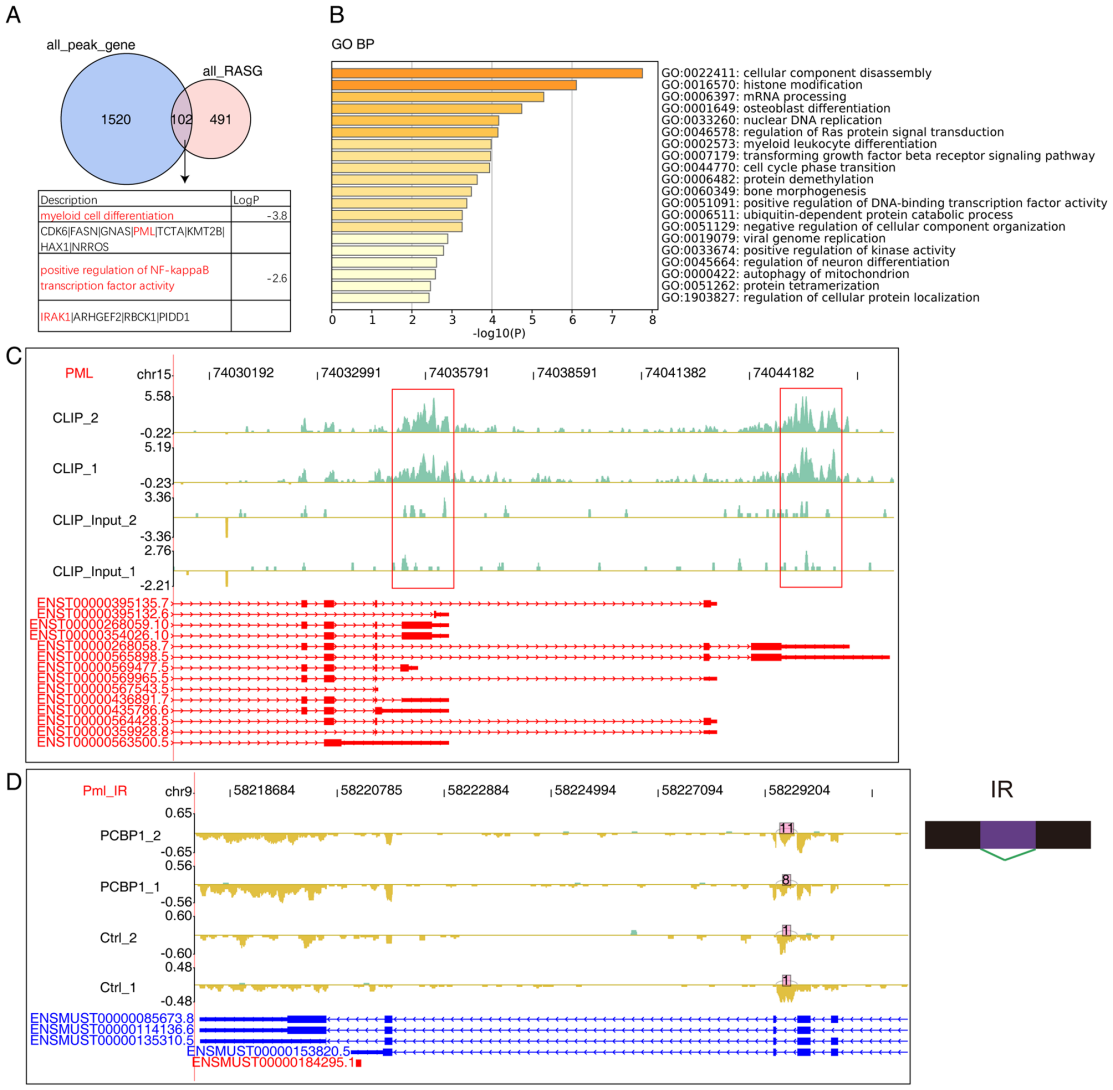
PCBP1 selectively binds to mRNA to regulate alternative splicing. (**A**) Venn diagram showed the overlap of PCBP1-bound peak’s genes and PCBP1-regulated alternatively splicing genes, and the PCBP1-bound peak’s genes is the union of two IP samples. LogP: log2 pvalue of enrichment terms. (**B**) Bar plot exhibited the most enriched GO biological process results of the overlap peak’s genes in figA, that making use of analyses by Metascape. (**C**) IGV-sashimi plot showed the UPF1-bound sites across mRNA of PML. The transcript of the gene was plotted at the bottom of the graph. (**D**) IGV-sashimi plot showed the UPF1-regulated alternative splicing events across mRNA of Pml. The transcript of the gene was plotted at the bottom of the graph and on the right was a model diagram of splicing events.

Based the hypothesis that PCBP1 regulates alternative splicing by directly binding to the pre-RNA transcripts, we made an extensive analysis between *Pcbp1*-KD RNA-seq and PCBP1 CLIP-seq datasets. We first analyzed the overlapped genes between PCBP1-bound genes and RASGs in RNA-seq, and obtained 102 such genes, showing significant interaction between PCBP1-bound genes and RASGs (Fig. 5A *P*-value = 7.173462e−26, Hypergeometric test). Functional enrichment analysis of these co-regulated genes showed they were enriched in cellular component disassembly, histone modification, osteoblast differentiation, nuclear DNA replication, regulation of Ras protein signal transduction, and other pathways (Fig. 5B, top 20 pathways by significance). Meanwhile, we also detected two pathways highly associated with immune and inflammatory response: myeloid cell differentiation and positive regulation of NF-κB transcription factor activity^27, 28^. Eight genes were included in myeloid cell differentiation pathway: *CDK6*, *FASN*, *GNAS*, *PML*, *TCTA*, *KMT2B*, *HAX1*, and *NRROS*. Positive regulation of NF-κB transcription factor activity included four genes: *IRAK1*, *ARHGEF2*, *RBCK1*, and *PIDD1* (Fig. 5C). We observed abundant PCBP1 binding signals within *PML* genomic locus, especially in exon and 3′UTR regions (Fig. 5C, red rectangular frame). Alternative splicing analysis revealed an IntronR event was reduced in *Pcbp1* deficiency Th1 cells, which was occurred adjacent to PCBP1 binding sites (Fig. 5D), suggesting that PCBP1 regulates *PML* alternative splicing by directly binding to its pre-RNA transcript. We also noticed *IRAK1* in NF-κB transcription factor pathway. PCBP1-bound peak was identified at exon5-exon6 region of *IRAK1* compared with input samples (Fig. S4A), and there was no significant difference except this region between CLIP and input samples. We also found a cassette exon event at the same region of *IRAK1* in mouse genome (Fig. S4B), supporting the conclusion that PCBP1 binding regulates alternative splicing of genes involved in the pathogenesis of RA.

### Experimental validation on genes regulated by PCBP1 in specimens from RA

**Figure 6:**
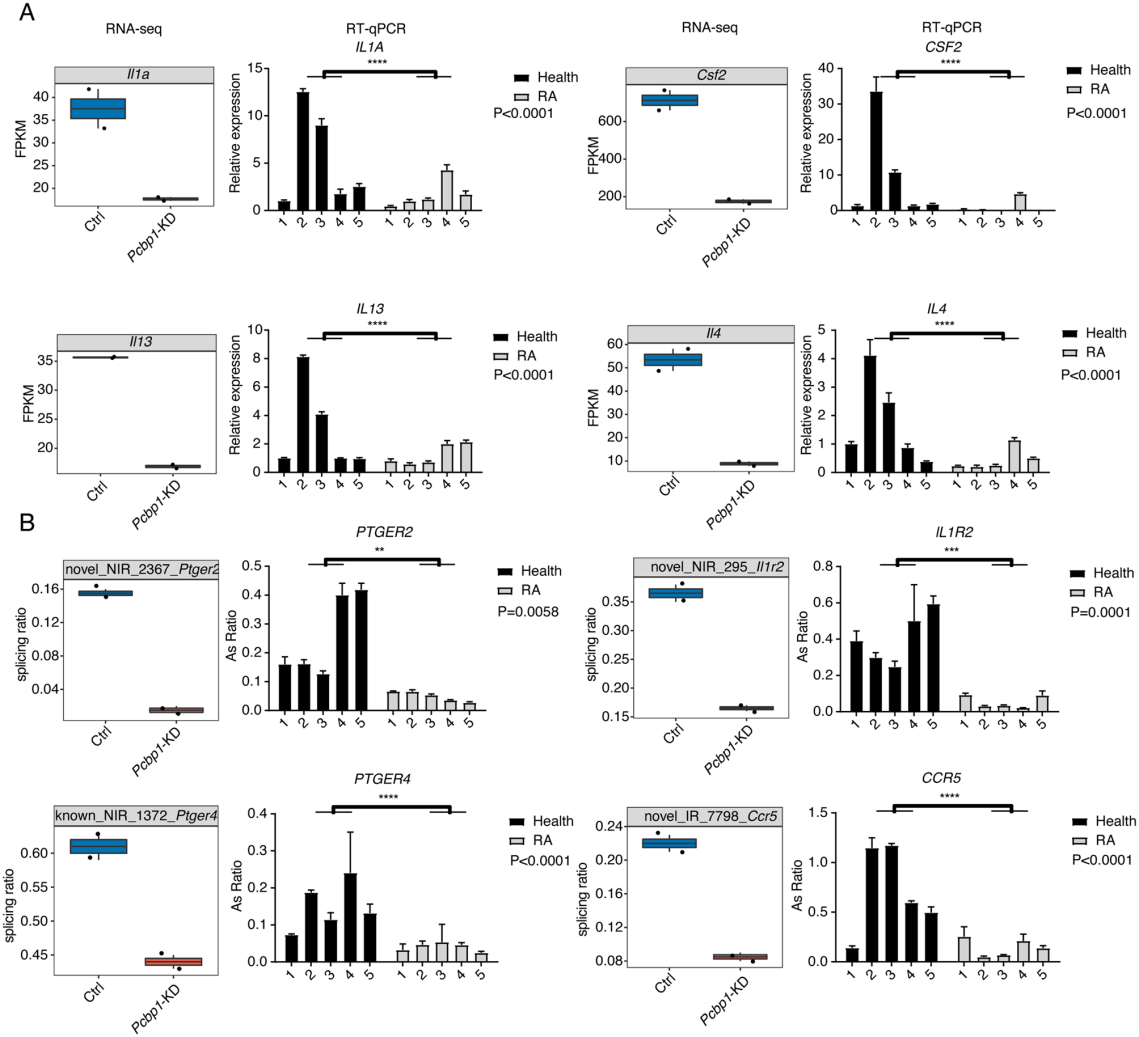
Experimental validation on DEGs RASGs by PCBP1 in PBMCs specimens from RA patients. (**A**) Bar plot showing the RT-qPCR validation results of immune and inflammation related DEGs regulated by PCBP1 in PBMCs from RA. Box plot in the left panel was expression level from RNA-seq in mice, and right panel was RT-qPCR result from RA patients. (****P-value < 0.0001; two-ways ANOVA test). (**B**) Bar plot showing the RT-qPCR validation results of immune and inflammation related RASEs regulated by PCBP1 in PBMCs from RA. Box plot in the left panel was AS ratio from RNA-seq in mice, and right panel was RT-qPCR result from RA patients. (**P-value < 0.01; ***P-value < 0.001; ****P-value < 0.0001; two-ways ANOVA test).

We have noticed PCBP1 mRNA level was downregulated in PBMCs specimens of RA patients from the first part, suggesting its potential regulatory roles in PBMCs. We thus want to make an experimental validation on PCBP1-regulated DEGs and AS genes identified above. Five RA patients and five normal controls with matched age and sex features were involved to extract PBMCs and following experiments. Four genes from immune and inflammatory response pathway, including *IL1A*, *CSF2*, *IL13*, and *IL4*, were selected as candidates; the results showed all of them were significantly downregulated in RA patients, showing high consistency with the RNA-seq data obtained from mouse Th1 cells, although there was individual variation within the same group (Fig. 6A). We also selected RASEs from genes enriched in immune and inflammation pathways, including *PTGER2*, *IL1R2*, *PTGER4*, and *CCR5*, and all of them were consistently validated with their changed AS ratios from the RNA-seq data (Fig. 6B). These results together demonstrate that PCBP1-regulated genes in mice are also changed in PBMC specimens from RA patients, suggesting the regulatory functions of PCBP1 in RA patients.

## Discussion

RA is a chronic, inflammatory, autoimmune disease characterized by persistent synovitis that primarily affects the joints and leads to poor quality of life. Although the etiology of RA is not fully understood, the imbalance between T helper cell subsets, including Th1/Th2 and Th17/Treg balance, is thought to play a key role in the initiation and perpetuation of the RA^16^. Among these T helper cell subsets, Th1 cells can promote the development of a pro-inflammatory microenvironment in the synovium by inducing the secretion of pro-inflammatory cytokines^29, 30^, which ultimately leads to bone erosion and cartilage destruction. Hence, RA is known to be a Th1-mediated autoimmune disorder, and therapies targeting Th1 cells could improve the treatment outcomes among RA patients. It is well known that iron deposition can be observed in the synovial fluid from RA patients^7–9^. Previous studies showed that both iron overload^31^ and iron deficiency^32^ could affect Th1 cells function by altering a variety of processes. In fact the iron chaperone PCBP1 is a critical mediator for maintaing iron homeostasis^13, 14^ and participates in the regulation of immune response. Wang et al.^19^ reported that *Pcbp1* deficiency inhibited pro-inflammatory cytokine expression in Th1 cells by inhibiting GM-CSF production and affecting *Csf2* RNA stability. However, the roles of PCBP1 in RA remain unknown, and the molecular mechanisms on how PCBP1 regulates Th1 cells to participate in RA are unclear.

Xia et al.^20^ previously tested the PCBP1 protein levels in plasma by ELISA assay, and showed that RA patients have significantly lower levels of PCBP1 compared with controls. Consistent with the result, we also found that the relative expression levels of *Pcbp1* in RA group were significantly lower than those of control group based on two public microarray datasets, providing the solid evidence that PCBP1 is involved in pathogenesis of RA. A large number of datasets suggest that PCBP1 can regulate the transcription of multiple genes^33^. We investigated the effect of PCBP1 silence in Th1 cells on transcriptional profile. The *Pcbp1* silencing in Th1 cells affected a total of 1899 genes, 1231 of which were up-regulated, and 668 down-regulated. Of note, these down-regulated DEGs were enriched in inflammatory response related terms, including cytokine production involved in immune response, inflammatory response, cytokine-mediated signaling pathway, and IL-1α production, which are closely related to RA^2, 34, 35^. Taken together, these results indicated that PCBP1 probably participates in the pathogenesis of RA via affecting the expression of genes involved in immune and inflammatory response in Th1 cells.

In addition to transcription regulation, as a canonical RBP, PCBP1 are also involved in post-transcriptional regulation, such as alternative splicing of pre-mRNA, which is an important post-transcriptional regulatory mechanism with more than 90% mammalian gene transcripts undergoing^36^. Increasing evidences have been found on the link between RA and the aberrant expression of splicing factors. Muller et al.^37^ demonstrated that folylpolyglutamate synthetase pre-mRNA splicing alterations could impact responsiveness of low dose methotrexate therapy for RA patients. Turkkila et al.^24^ also reported that suppressed diversity of alternative splicing of survivin occurred in active RA. In the present study, we found that knockdown of *Pcbp1* affected not only transcription but also alternative splicing levels in Th1 cells. A total of 2128 RASEs were significantly regulated by PCBP1 in Th1 cells, including 424 down-regulated IntronR events. However, the overlap (146 genes) between DEGs and RASGs was very low (6.9% in ASGs, 7.7% in DEGs), suggesting that majority of mRNA alternative splicing and differential expression events were regulated by PCBP1 independently. In one previous study, Wang et al.^18^ showed that *Pcbp1* deficiency could affect *Csf2* RNA stability, thereby inhibiting pro-inflammatory cytokine expression in Th1 cells. It is interesting to explore whether PCBP1 regulates the inflammatory response in Th1 cells by alternative splicing. In our study, enrichment analysis showed that some PCBP1-RASGs occurred in TNF-a/NF-κB pathway, T cell activation, T cell differentiation and T cell differentiation associated immune response pathways, which were well known to be associated with occurrence and development of RA. These findings suggest that PCBP1 may play a part in RA pathogenic mechanism by modulating alternative splicing of genes involved in immune response in Th1 cells. Meanwhile, we also noticed one limitation of our study is that the discovery is not fully validated in human samples with RA by silencing or enhancing PCBP1 expression, such as naïve T cells from RA patients. The RT-qPCR experiments for DEGs and RASGs in this study can partly make up this shortage and validate the regulatory relationship between PCBP1 and its targets, further studies are also needed to further investigate the underlying mechanisms with more samples.

To assess its direct role in the regulation of alternative splicing, genome-wide RNA-binding profile of PCBP1 was analyzed by CLIP-seq, which usually generates short-length reads and identifies exact binding sites. Consistent with the previous reports^18, 38, 39^ that PCBP1 regulated the stability of multiple mRNAs by binding to CU-rich elements in 3′UTRs, the genomic distribution and motif analyses of the overlapped peaks from the two CLIP samples also revealed that PCBP1 primarily bound to the CU-rich elements in 3′UTRs of protein-coding transcripts in Jurkat T cells. In addition, we obtained 102 overlapped genes between PCBP1-bound genes and RASGs, showing significant interaction between PCBP1-bound genes and RASGs. It is notable that these genes were predominantly enriched in several immune response related pathways, including myeloid cell differentiation and positive regulation of NF-κB transcription factor activity. Myeloid cells are of critical importance in the initiation and perpetuation of synovitis in RA. They can recruit and promote the differentiation of T cells into inflammatory phenotypes in RA synovium^40, 41^. Rencently, Yan et al.^42^ reviewed the effects of myeloid-derived suppressor cells (MDSCs) on RA and reported that MDSCs have both anti-inflammatory and pro-inflammatory effects during the development of RA. As a critical regulator in myeloid cell differentiation pathway^43, 44^, PML belongs to the the TRIM family, and has been demonstrated to be involved in diverse disorders, including primary biliary cholangitis^45^, viral infections^46^, cancers^47^, and as well as RA^48^. We found that PCBP1 could regulate *PML* alternative splicing in Th1 cells by directly binding to its pre-RNA transcript. Moreover, another intriguing finding of the present study is that PCBP1 can regulate alternative splicing of *IRAK1* invovled in NF-κB transcription factor pathway. IRAK1 has been well knonwn to be asscociated with occurrence and development of RA. Numerous studies have showed that the polymorphism of *IRAK1* can increase the risk and the severity of RA^49, 50^. These findings give us a hint that PCBP1 can regulate alternative splicing of known RA-related genes, which would be worthwhile to be further explored in future.

## Conclusion

To our best knowledge, this is the first study on how PCBP1 participates in RA. By performing integrated data analysis of *Pcbp1*-knockdown RNA-seq and PCBP1 CLIP-seq, our study suggests that PCBP1 probably participates in the molecular mechanism of RA via not only affecting the expression of genes involved in immune and inflammatory response in Th1 cells, but also binding to and regulating the alternative splicing of immune response related genes in Th1 cells. In light of this understanding, PCBP1 may become an effective therapeutic target for RA in the future. However, much experimental work should be carried out to definite the functions of PCBP1 in RA.

## Materials and methods

### Retrieval of public data

To investigate the *Pcbp1* expression changes in patients with RA, two microarray datasets of GSE15573 (stored by Olaso et al.^51^) and GSE23561 ((deposited by Grayson et al.^52^) were downloaded from the Gene Expression Omnibus (GEO) database (https://www.ncbi.nlm.nih.gov/geo/). GSE15573 contians 33 peripheral blood mononuclear cells (PBMCs) samples from 18 RA patients and 15 controls, and the platform is Illumina human-6 v2.0 expression beadchip. As for the dataset GSE23561, it includes 6 RA-PBMCs samples and 9 control-PBMCs samples, and its platform is Human 50 K Exonic Evidence-Based Oligonucleotide array. To further decipher the molecular mechanisms of PCBP1 in RA, we also downloaded published dataset of GSE84702 (deposited by Wang et al.^18^), which includes the *Pcbp1* deficiency RNA-seq data in mouse Th1 cells and the sequencing data of PCBP1 crosslinking and immunoprecipitation (CLIP-seq) in Jurkat T cells.

### Microarray data processing and relative levels of PCBP1 analysis

The microarray data probe was transformed to gene symbols. If several probes were mapped to one gene symbol, the mean value was set as the final expression value of this gene. We use online GEO2R with default parameters (https://www.ncbi.nlm.nih.gov/geo/geo2r/) to compare two or more groups of samples in order to identify genes that are differentially expressed across experimental conditions. Adjusted *P*-value < 0.05 and |log2fold change (FC)|> = 1 were chosen as the cut-off thresholds.

### RNA-seq data processing and differential expression of genes (DEGs) analysis

The raw reads were trimmed of adaptors and low-quality bases using a FASTX-Toolkit (v.0.0.13; http://hannonlab.cshl.edu/fastx_toolkit/). Then, the clean reads were evaluated using FastQC (http://www.bioinformatics.babraham.ac.uk/projects/fastqc/) and were aligned to the mouse GRCm38 genome usingTopHat2^53^ with 4 mismatches. After mapping reads onto the genome, we discarded the reads with multiple genomic locations due to the ambiguous origination. Reads with only one genome location were preserved to calculate read number and FPKM value (FPKM represents fragments per kilobase and per million mapped) for each gene. The differential expression of genes (DEGs) were analyzed by the software edgeR^54^. The results were analyzed based on the fold change (FC ≥ 2 or ≤ 0.5) and false discovery rate (FDR ≤ 0.05) to determine whether a gene was differentially expressed.

### RNA-seq data alternative splicing analysis

The alternative splicing events (ASEs) and PCBP1 regulated alternative splicing events (RASEs) between the samples were defined and quantified by using the ABLas pipeline as described previously^55, 56^. In brief, ABLas detection of ten types of ASEs was based on the splice junction reads, including exon skipping(ES), alternative 5′splice site (A5SS), alternative 3′splice site (A3SS), intron retention (IntronR), mutually exclusive exons (MXE), mutually exclusive 5′UTRs (5pMXE), mutually exclusive 3′UTRs (3pMXE), cassette exon, A3SS&ES and A5SS&ES. For sample pair comparison, Fisher’s exact test was selected to determine statistical significance, using the alternative reads and model reads of the samples as input data. We calculated the changed ratio of alternatively spliced reads and constitutively spliced reads between compared samples, which was defined as the RASE ratio. The RASE ratio ≥ 0.2 and *P*-value ≤ 0.05 were set as the threshold for RASEs detection. For repetition comparison, Student’s *t*-test was performed to evaluate the significance of the ratio alteration of AS events. Those events which were significant at *P*-value cutoff of 0.05 were considered RASEs.

### CLIP-seq data processing and analysis

For CLIP-seq data, data processing method was the same to the RNA-seq data. The clean reads were aligned to the human-GRCh38 genome by STAR^57^. Followed by only uniquely mapped reads were used for the following analysis. “ABLIRC” strategy was used to identify the binding regions of RNA binding protein on genome^55^. Reads with at least 1 bp overlap were clustered as peaks. For each gene, computational simulation was used to randomly generated reads with the same number and lengths as reads in peaks. The outputting reads were further mapped to the same genes to generate random max peak height from overlapping reads. The whole process was repeated for 500 times. All the observed peaks with heights higher than those of random max peaks (*P*-value < 0.05) were selected. The immunoprecipitation and input samples were analyzed by the simulation independently, and the IP peaks that have overlap with input peaks were removed. The target genes of IP were finally determined by the peaks and the binding motifs of IP protein were called by HOMER software^58^.

### Pathway and process enrichment analysis

For each given gene list, pathway and process enrichment analysis has been carried out with the following ontology sources: Gene Ontology (GO) Biological Processes, Kyoto Encyclopedia of Genes and Genomes (KEGG) Pathway^59, 60^, Reactome Gene Sets, CORUM, TRRUST, PaGenBase and WikiPathways^61^. All genes in the genome have been used as the enrichment background. Terms with a *P*-value < 0.01, a minimum count of 3, and an enrichment factor > 1.5 (the enrichment factor is the ratio between the observed counts and the counts expected by chance) are collected and grouped into clusters based on their membership similarities. More specifically, *P*-values are calculated based on the accumulative hypergeometric distribution, and *q*-values are calculated using the Banjamini-Hochberg procedure to account for multiple testings.

### Validation of gene expression in RNA-Seq by qRT-PCR analysis

To evaluate the validity of the PCBP1-regulated DEGs and AS genes in RNA-seq data, qRT-PCR was performed. Whole blood samples were obtained from 5 RA patients and 5 age-and-gender-matched healthy controls in our hospital. All the blood samples were processed immediately after collection for the isolation of peripheral blood monouclear cells (PBMCs). Total RNA was extracted from PBMCs using the TRIzol reagent (Invitrogen) according to the manufacturer’s instructions. Then, the purified RNA was reverse-transcribed taken for complementary DNA by PrimeScript RT reagent Kit (Takara). Subsequently, qRT-PCR was conducted by using TB Green Fast qPCR Mix (Takara) and specific primers (Supplementary Table). ACTB (Beta-actin) was used as a control gene for assessing the relative expression of DEGs. PCR amplifications were quantified using 2^−ΔΔCT^ method. Two-ways ANOVA test was carried out to determine the expression difference between RA and control group.

### Ethics approval and consent to participate

The Ethics Committee of Henan Provincial People’s Hospital approved the study (#HNSRMYY-2020-138). All subjects volunteered for the study and signed informed consent forms prior to sample collection. We have strictly kept the standard biosecurity and institutional safety procedures in our country and area (Biosecurity Law of People’s Republic China). And all methods were performed in accordance with the Declaration of Helsinki.

## Supplementary Information

Supplementary Figure S1.

Supplementary Figure S2.

Supplementary Figure S3.

Supplementary Figure S4.

Supplementary Legends.

Supplementary Table 1.

## Data Availability

The microarray datasets that support the findings of this study are openly available in the Gene Expression Omnibus (GEO) database (https://www.ncbi.nlm.nih.gov/geo/). The two public gene expression data sets, GSE15573, and GSE23561 can be downloaded from NCBI Gene Expression Omnibus database at (https://www.ncbi.nlm.nih.gov/geo/).
