# Supplementary figures and images for: PCBP1 is associated with rheumatoid arthritis by affecting RNA products of genes involved in immune response in Th1 cells
Source: Sci Rep. 2022 May 19;12:8398. doi: 10.1038/s41598-022-12594-7 (PMC9120163; doi:10.1038/s41598-022-12594-7)

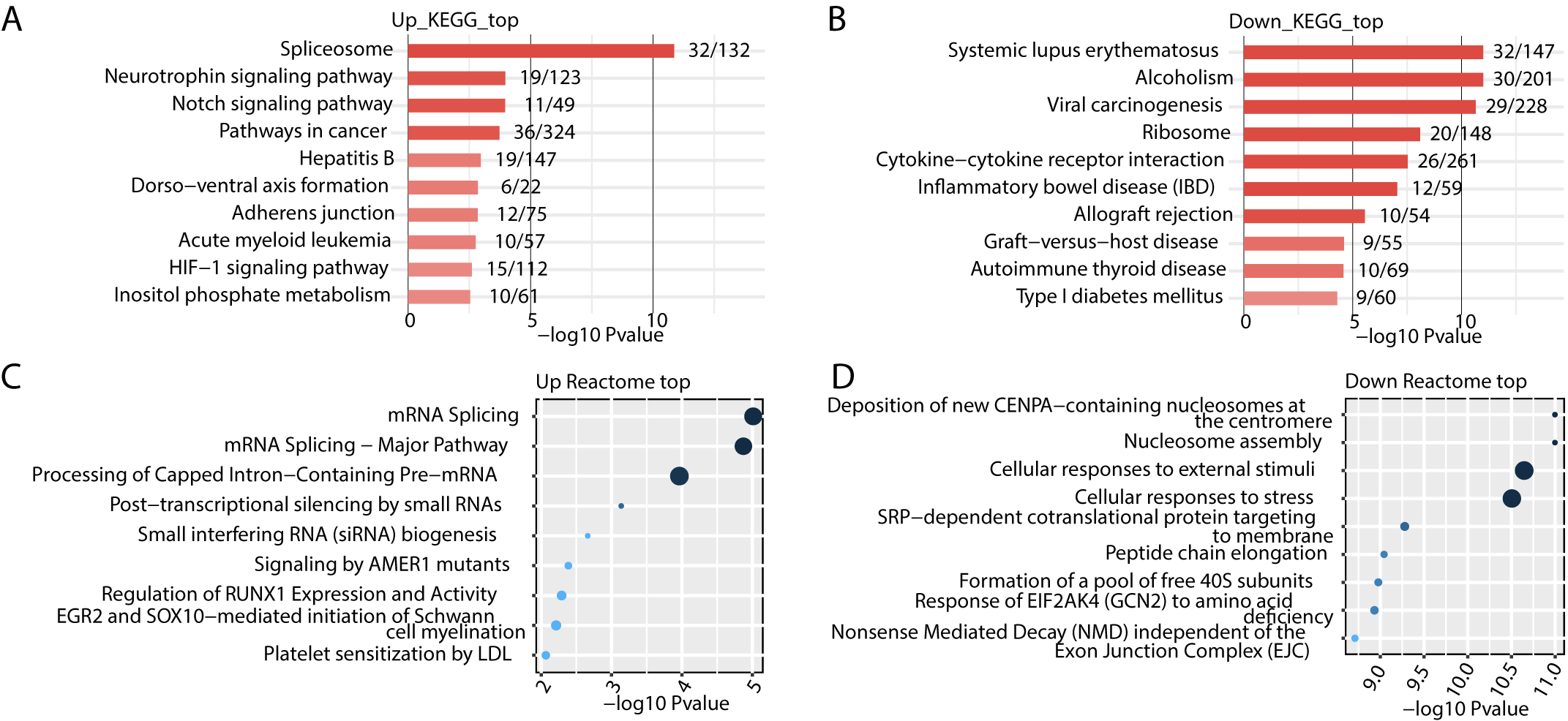

Supplement: Supplementary file 1 — Supplementary Figure S1. [file 41598_2022_12594_MOESM1_ESM.tif]

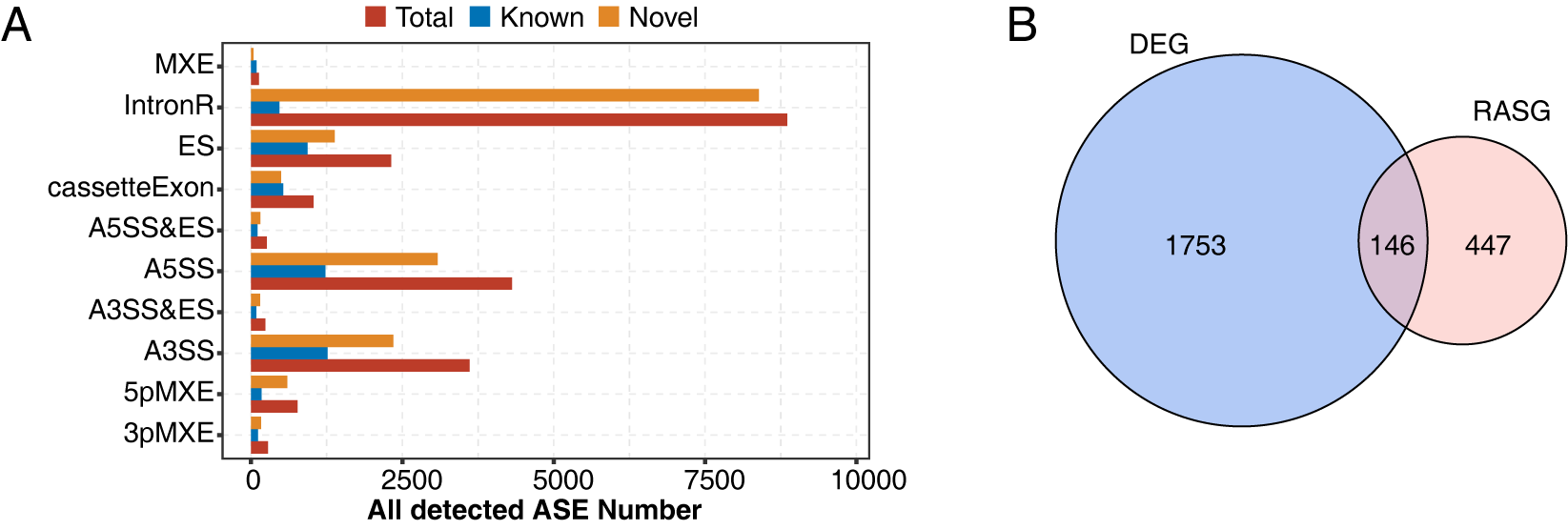

Supplement: Supplementary file 2 — Supplementary Figure S2. [file 41598_2022_12594_MOESM2_ESM.tif]

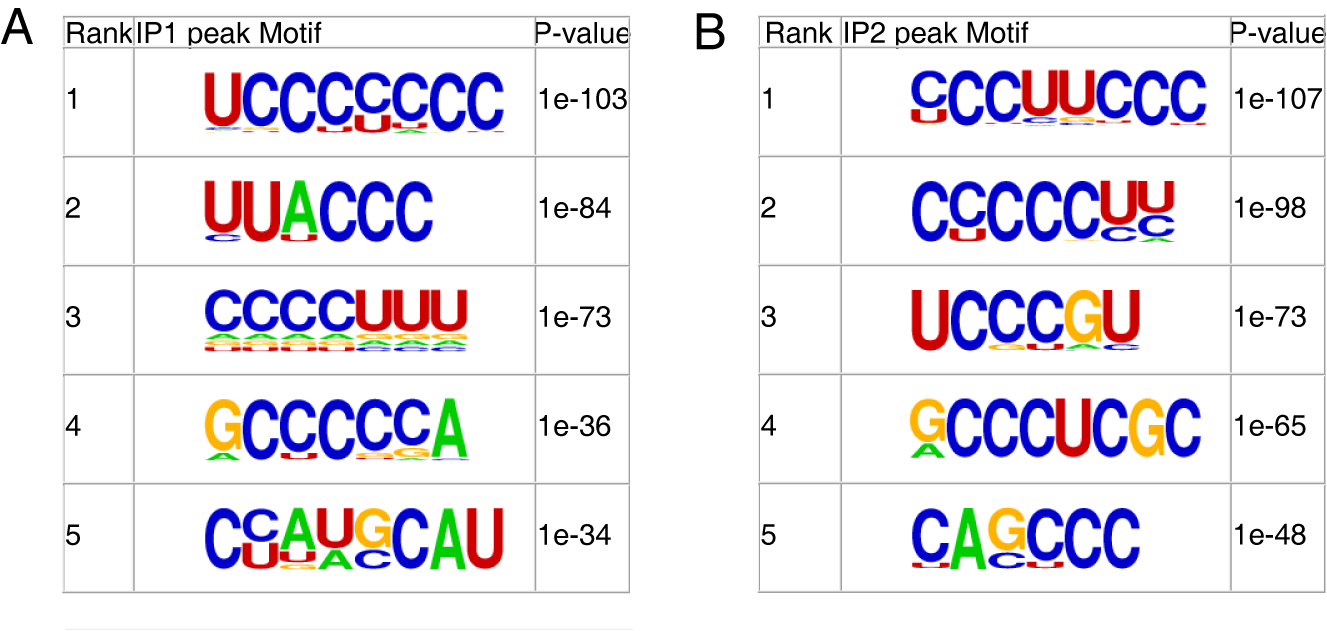

Supplement: Supplementary file 3 — Supplementary Figure S3. [file 41598_2022_12594_MOESM3_ESM.tif]

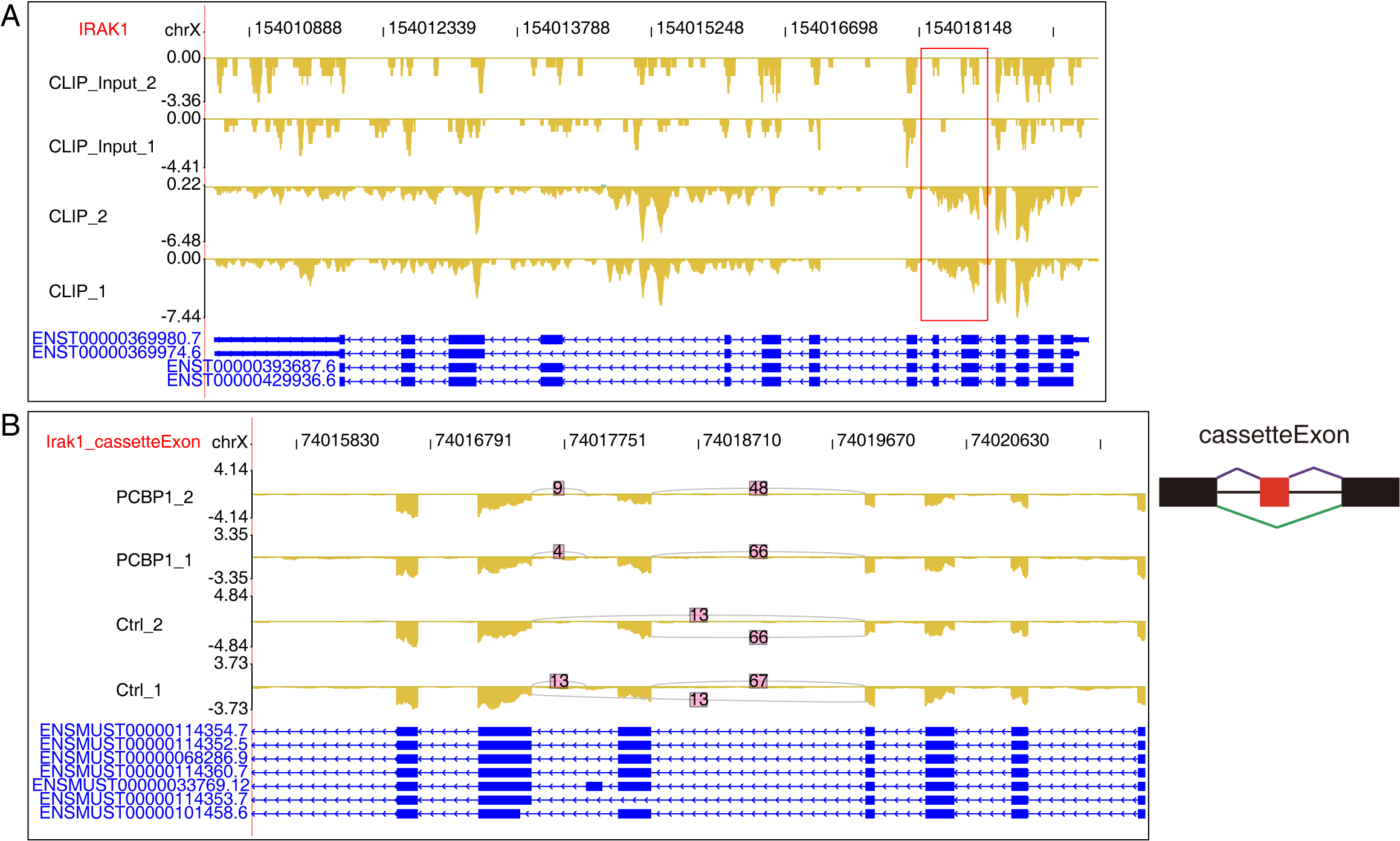

Supplement: Supplementary file 4 — Supplementary Figure S4. [file 41598_2022_12594_MOESM4_ESM.tif]
