# Supplementary material for: PCBP1 is associated with rheumatoid arthritis by affecting RNA products of genes involved in immune response in Th1 cells
Source: Sci Rep. 2022 May 19;12:8398. doi: 10.1038/s41598-022-12594-7 (PMC9120163; doi:10.1038/s41598-022-12594-7)
Supplement: Supplementary file 5 — Supplementary Legends. [file 41598_2022_12594_MOESM5_ESM.pdf]

Figure S1

Figure S1. Transcriptome analysis of differentially expressed genes (DEGs) in *Pcbp1*-knockdown Th1 cell and control Th1 cell.

A. Bar plot exhibited the most enriched KEGG pathways results of the up-regulated DEGs.

B. Bar plot exhibited the most enriched KEGG pathways results of the down-regulated DEGs.

C. Scatter plot exhibited the most enriched Reactome pathways results of the up-regulated DEGs.

D. Scatter plot exhibited the most enriched Reactome pathways results of the down-regulated DEGs.

Figure S2

Figure S2. Transcriptome analysis of alternative splicing regulation in *Pcbp1*-knockdown and control samples.

A. The bar plot showed the number of all detected alternative splicing events (ASEs). X-axis: ASE number. Y-axis: the different types of AS events.

B. Venn diagram showed the overlap genes number of DEGs and RASGs.

Figure S3

Figure S3. CLIP-seq analysis revealed the RNA binding features of PCBP1 in T lymphoma cell line.

A. Motif analysis showed the top 5 preferred bound motifs of PCBP1 in IP1 sample by HOMER software.

B. Motif analysis showed the top 5 preferred bound motifs of PCBP1 in IP2 sample by HOMER software.

Figure S4

Figure S4. PCBP1 selectively binds to mRNA to regulate alternative splicing.

A. IGV-sashimi plot showed the UPF1-bound sites across mRNA of IRAK1. The transcript of the gene was plotted at the bottom of the graph.

B. IGV-sashimi plot showed the UPF1-regulated alternative splicing events across mRNA of *IRAK1*. The transcript of the gene was plotted at the bottom of the graph and on the right was a model diagram of splicing events.
